# On the Looming Physician Shortage and Strategic Expansion of Graduate Medical Education

**DOI:** 10.7759/cureus.9216

**Published:** 2020-07-15

**Authors:** Harris Ahmed, J. Bryan Carmody

**Affiliations:** 1 Ophthalmology, Loma Linda University Medical Center, Loma Linda, USA; 2 Pediatrics, Eastern Virginia Medical School, Norfolk, USA

**Keywords:** graduate medical education, residency, physician shortage, medicare, gme

## Abstract

Among many other things, the novel coronavirus pandemic of 2020 highlighted the significance of physician shortages in the United States. Current projections anticipate a national shortage of up to 122,000 physicians by 2032, with shortfalls in both primary care physicians and specialists. Yet while this figure highlights the magnitude of the problem, it does not capture the distributional aspect of American physician shortages. Though some specialties and geographic areas have a surplus of physicians, others have a chronic undersupply. Appropriately addressing the looming physician shortage therefore requires not only creating more physicians, but also ensuring that those physicians practice in the areas of greatest societal need. This review explores the nature of physician shortages in the United States, identifies the present bottleneck in physician training at the level of graduate medical education, and considers potential legislative and policy solutions to allow strategic and deliberate expansion of graduate medical education and physician practice.

## Introduction and background

The 2020 novel coronavirus (COVID-19) pandemic highlighted the significance of physician shortages in the United States. Both physicians and the public have been jarred by accounts of severe staffing issues in areas hit hardest by COVID-19. Some states, such as New York, have taken exceptional measures to get physicians into the positions in which they are needed, including redeployment of specialists, early graduation for medical students, return of retired practitioners, and relaxation of state licensure regulations to allow out-of-state physicians to assist higher need areas. Meanwhile, in states less impacted by COVID-19, physicians have been furloughed as clinics and operating rooms sat empty. Doctor shortages and regional variations in physician supply existed before COVID-19 and will continue to exist after, unless we seize the opportunity for thoughtful and comprehensive reform.

The Association of American Medical Colleges projects that the United States will see a shortage of up to 122,000 physicians by 2032, with demand exceeding supply not only for primary care, but also specialist physicians [1]. While these raw figures highlight the magnitude of the problem, they fail to capture the distributional aspect of physician shortages. Two decades ago, the Council on Graduate Medical Education cited the geographic maldistribution of health care as “the central paradox in the American healthcare system: shortages amid surplus” [2]. This paradox persists today, and any plan to address the looming physician shortage must directly confront this issue.

## Review

The nature of the problem

Evidence of the geographical maldistribution of physicians is apparent at whatever scale geography is considered. When classified by United States (U.S.) Census region, the South has the greatest current need for physicians (31,000 physicians), while the Northeast may already be oversupplied by nearly 24,000 physicians [1]. If the physician workforce is considered within individual states, or by individual specialties rather than total physicians, disparities persist. For instance, the five states with the greatest number of hand surgeons per capita have three times as many surgeons as the five states with the fewest [3]. Similarly, while there are 3.4 pediatric nephrologists per 100,000 children in Vermont, in Wyoming, Montana, and North Dakota there are none [4]. Even within the same state there can be immense variation in physician distribution. For example, Santa Monica, California has 35.9 dermatologists per 100,000 people, while just an hour’s drive away in Mojave, there are only 0.38 dermatologists per 100,000 people [5].

Yet even within the same city, there can be dramatic variation in geographic access to care. For example, in Philadelphia, there is no net primary care physician shortage, nor is the city designated as a health professional shortage area. However, the supply of primary care physicians varies significantly across census tracts within the city, ranging between 105 and 1,032 among various census tracts. Low physician access census tracts were more likely to be in a neighborhood with a higher proportion of African Americans, even after adjusting for socioeconomic and insurance status [6].

The graduate medical education bottleneck

Importantly, while the United States may face a future shortage of *physicians*, it does not presently have a shortage of *doctors* [7]. This distinction is important. Annually, over 4,000 American citizens graduate as doctors from allopathic, osteopathic, and international medical schools, but are unable to obtain residency training positions in the United States [7]. Yet without residency training, these doctors are unable to become licensed physicians capable of serving the public. All U.S. states require at least one year of postgraduate residency training for physician licensure, and many states require more (particularly for graduates of international medical schools) [8].

The primary bottleneck in the physician pipeline, therefore, is the presence of graduate medical education (GME) positions in disciplines that match future workforce needs. Increasing the physician supply will require expanding GME. However, the availability of GME training positions is constrained by financing. 

The manner in which GME positions are funded has evolved over the past century. Until the 1940s, hospitals paid resident stipends by building these costs into patient charges [9]. However, after World War II, the federal government began to increasingly subsidize resident training. The 1965 Medicare Act established broad federal funding for resident education, though this funding was intended to be temporary. As noted in the House and Senate reports, “[I]t is intended, until the community undertakes to bear such educational costs in some other way, that part of the net cost of such [educational] activities (including stipends of trainees, as well as compensation of teachers and other costs) should be borne to an appropriate extent by the hospital insurance program” [9]. Because Medicare still accounts for the majority of GME funding (nearly $10 billion of the $15 billion annual costs), the overall growth in GME has been constrained by the implementation of the 1997 Balanced Budget Act, which capped the number of residents that could receive direct GME funding from the Centers for Medicare and Medicaid Services (CMS) [10].

A half-measure: lifting the Medicare direct medical education cap

Some organizations, such as the American Association of Medical Colleges and American Association of Colleges of Osteopathic Medicine, have called for an end to the CMS cap on direct GME expenses as a way to ease the bottleneck between the number of doctors graduating medical school and the number of physicians able to serve the public [1,11]. Such a move is logical: the physician workforce cannot be increased without expanding GME. Moreover, the availability of GME positions indirectly influences practice geography, since over half of physicians remain in the state where they completed their GME [12]. Yet while additional funding for GME will be necessary, in isolation, such a solution does not adequately address geographic and specialty maldistributions. Instead, effectively dealing with the physician shortage requires strategic or directive GME creation in order to target geographic areas and specialties of highest need and prevent continued oversaturation of GME rich regions.

Consider how GME has grown since 1997. A Government Accountability Office (GAO) report found that from 2005 to 2015, the number of residents grew by 22%, but the geographic regions where residents trained remained largely unchanged [13]. Although primary care positions increased, growth in subspecialties occurred more than twice as rapidly. Moreover, the geographic locations of GME positions remained largely unchanged despite uneven population growth across regions. Lastly, the GAO noted that CMS funding for GME does not target federally designated workforce needs areas. 

Put another way, even without additional federal subsidies, hospitals have increased the number of GME positions available. Yet rather than expanding to address societal needs as part of a comprehensive policy, the individual decisions to expand GME positions were made in response to local market forces. For a hospital, the financial benefit of adding a residency position is variable. A comprehensive analysis by the RAND Corporation demonstrated that the impact of adding a single additional internal medicine resident in 2010 could vary from an annual $100,000 loss to a greater than $250,000 financial gain for the institution, depending on the balance between revenue generated and cost of replacement [14]. In general, adding a resident in subspecialty, procedural, or heavily inpatient residency programs results in greater financial gains for hospitals, given the additional patient care revenue that can be created and the increased marginal cost of creating this revenue with a non-resident provider. Conversely, adding a resident in primary care or predominantly outpatient specialties generates fewer financial efficiencies for the sponsoring hospital [14]. 

Indiscriminately lifting the CMS direct GME cap would likely result in hospitals adding residency positions that would not have been financially viable without federal subsidy. Yet how many of these positions will be in the specialties most needed by patients, or would result in training physicians that will ultimately practice in high need areas, is uncertain. Without a more specific incentive to train doctors in the areas and disciplines that mirror the needs of society, local market forces will reign, and hospitals will continue to create residency positions primarily based upon the marginal benefit to the hospital’s bottom line.

Opportunities for legislative reform

Several recent legislative proposals could achieve the goals of directive and strategic GME expansion. For example, the Resident Physician Shortage Reduction Act of 2019 (S. 348/H.R. 1763) would increase physician training spots nationally by 15,000 over five years, and would additionally prioritize residency positions for hospitals in states with new medical schools or new branch campuses and emphasize training in community-based settings. Another proposal, the Rural Physician Workforce Production Act of 2019 (S. 289), would expand rural medical residency training programs by establishing comparable per-resident payment for training in rural hospitals relative to those in urban communities and eliminate rural hospital residency caps to encourage growth of rural training programs. Lastly, the Supporting Graduate Medical Education at Community Hospitals Act of 2019 (S. 2116/H.R.3753) would allow more programs to create new caps and establish new per-resident amounts.

There are additional opportunities for legislative initiatives to address the distributional aspects of physician shortages. Programs such as the Teaching Health Center Graduate Medical Education have retained nearly 60% of their graduates in rural or medically underserved areas and could provide billions in savings for CMS [15]. Loan forgiveness for service programs (such as the Public Service Loan Forgiveness program) have also been effective at retaining physicians in high need and underserved areas [16,17].

Though inadequate as a single strategy, another potentially useful legislative strategy is to redistribute unused GME spots to high need areas and specialties. Some residency programs that received direct GME funding before the 1997 Balanced Budget Act cap have since closed or reduced the number of residents in the program, leaving their funded positions unused. These unused positions have been redistributed twice in the past. The first occurred in 2003 through the Medicare Prescription Drug, Improvement, and Modernization Act. Although over 3,000 GME positions were redistributed, rural hospitals received fewer than 3% of the new positions [18]. When GME redistribution occurred again as part of the 2010 Patient Protection and Affordable Care Act, 75% of unused positions were directed to primary care and general surgery programs, with priority to hospitals located in states with the lowest resident-to-population ratios or highest number of health professional shortage areas [19].

Re-examining government GME funding

Beyond lifting the cap on direct payments for GME, we should comprehensively consider the manner in which Medicare supports residency training and the incentive structure around it. The federal government contributes around $15 billion annually towards GME, with the majority of this funding ($9 billion) coming from Medicare [10]. Payment is provided not only for direct GME expenses (to cover resident salaries, faculty teaching, administration, and other costs of the residency program), but also for indirect medical expenses (IME). Theoretically, IME payments are intended to cover the hospital’s excess costs of care due to training residents, who may inefficiently order tests and care for patients with greater illness severity or longer length of stay. Although the majority of Medicare payments (70%) go toward IME, the Medicare Payment Advisory Commission found that only 40%-45% of these payments were empirically justified [20].

Both direct and IME payments are calculated based upon complicated formulae that were determined by CMS in the 1980s [21]. While the precise calculations are beyond the scope of this paper, the formulae make inpatient care more lucrative than focusing on community health and outpatient care [22]. Such a model is undesirable as health care shifts towards a preventive model that places primary care at the forefront. Indeed, some have suggested that capping IME at $150,000 per resident per year would result in $1.28 billion that could be redistributed to address other workforce needs [23].

However, not all governmental funds for GME come through Medicare. Other major GME funding entities include the Veterans Health Administration, the Department of Defense, and the Health Resources and Services Administration [10]. To achieve a truly comprehensive GME funding policy, these funds must be considered as part of the broader GME funding priorities rather than individual pieces that are evaluated only within the context of each group’s organizational priorities.

As the second largest source of GME funding in the United States (nearly $4B), Medicaid GME funding deserves special consideration. Currently, large differences in Medicaid funding patterns contribute to geographic disparities. Not all states provide GME funding through Medicaid, and among those that do, spending varies widely. In 2018, seven states provided over $200 million in GME payments under their Medicaid program, while another seven states provided no funding at all [24].

Other considerations

While this article has focused primarily on GME and the role of GME funding in addressing physician workforce shortages, a truly comprehensive policy must consider multiple other elements as well. For instance, the impact of a physician shortage could be attenuated without more physicians if policies were implemented that allowed existing practitioners to maximize their time and efficiency. Reducing paperwork and administrative burden would enable physicians to spend more time and see more patients. Conversely, allowing physicians to spend more and more time on electronic documentation will not only exacerbate the impacts of the physician shortage, but may also negatively impact patient outcomes [25].

A comprehensive plan also requires considering the role of non-physician providers, such as nurse practitioners and physician assistants. In fact, the most recent GAO report from 2019 considered diverting physician GME funding to training non-physician providers [26]. While non-physician providers can fill important gaps in the health care system, non-physician providers cannot replace physicians entirely or avert the physician shortage. Indeed, projections still predict a shortfall of physicians even in models that assume the highest demand for non-physician providers [1].

Physician supply in high need areas can also be increased by targeted medical school creation. Establishing medical schools in medically underserved areas is an effective way to retain physicians in a high need area since many medical students ultimately practice near where they attended medical school [12]. Osteopathic medical schools have been effective at establishing medical schools in high need areas, creating rural tracks, recruiting students from underserved communities, and placing graduates in high need specialties and areas [27]. Additionally, not only do osteopathic primary care physicians tend to practice in smaller cities and communities, but so do osteopathic surgical subspecialists [28,29]. Responsible and controlled growth of the osteopathic profession will contribute towards addressing geographic disparities. In general, medical schools (both allopathic and osteopathic) should enhance recruitment of underrepresented minorities as they are more likely to have intention to work with underserved populations [16].

Lastly, and perhaps most contentiously, physician compensation and financial incentives must be revisited. American medical students preferentially enter specialties that provide greater future compensation, and rising educational debt can provide a disincentive to students choosing lower-paying primary care specialties [30,31]. Providing financial compensation or expanding loan forgiveness programs for physicians entering practice in the most needed specialties or areas would create a powerful incentive to encourage doctors to work in the areas of greatest societal need. 

## Conclusions

The COVID-19 pandemic has highlighted the regional variation that exists in physician supply across the United States, and how these variations can negatively affect care especially in a crisis. Yet disparities in physician supply both predate and transcend the current pandemic, and these disparities will continue to exist until they are addressed by a thoughtful, deliberate, and comprehensive policy reform. To create a physician workforce in the numbers, and in the distribution of specialties and geography that patients need, we must act now to create a comprehensive GME policy.
